# Supplementary material for: Are carbon emissions trading and green financial instruments synergistic? -Comprehensive quantitative research based on content analysis
Source: PLoS One. 2024 Mar 7;19(3):e0298601. doi: 10.1371/journal.pone.0298601 (PMC10919601; doi:10.1371/journal.pone.0298601)
Supplement: S1 Data — (ZIP) [file pone.0298601.s001.zip › Data/地级市Z_F工作报告.zip!/╜¡╬≈/╛░╡┬╒≥╩╨/└┤╫╘╣1⁄2╓┌║┼í╛┬φ┐╦╩2╛▌═°í┐_2018.docx]

各位代表：

现在，我代表市人民政府向大会报告工作，请予审议，并请各位市政协委员和列席会议的同志提出意见。

一、2017年工作回顾

过去的一年，是景德镇历史上浓墨重彩、极不平凡的一年。我们认真学习贯彻党的十九大精神，以习近平新时代中国特色社会主义思想为指导，全面落实省委“创新引领、绿色崛起、担当实干、兴赣富民”工作方针和省政府各项工作部署，全力践行市委“三个五”战略行动，统筹推进城市、产业、文化、生态、民生等各项工作，较好地完成了市十五届人大二次会议确定的各项目标任务。预计全年实现生产总值900亿元，同比增长8.5%；财政总收入123.2亿元，增长6.4%；规模以上工业增加值增长8.8%；固定资产投资增长13.5%；社会消费品零售总额335亿元，增长12%；外贸出口52亿元，增长22.1%；实际利用外资2.05亿美元，增长9%；城镇居民人均可支配收入34246元，增长9%；农村居民人均可支配收入15127元，增长9%；单位生产总值能耗下降3.43%。

——这一年，我们收获了一个个国字号荣誉。获评国家森林城市；获批全国“城市双修”试点；入选全国首批26个通航产业综合示范区；进入第二批国家全域旅游示范区创建名录；获批设立“海峡两岸交流基地”。浮梁县被授予“全国十大魅力茶乡”称号，成为“全国休闲农业与乡村旅游示范县”。陶溪川列入首批10个国家级文化产业示范园创建资格名单，荣获2017联合国教科文组织亚太地区文化遗产保护创新奖。宇宙瓷厂列入第一批国家工业遗产认定名单。

——这一年，我们办成了一件件大事喜事。在德国G20峰会期间，配合中宣部在柏林成功举办“感知中国·匠心冶陶”景德镇陶瓷文化展，在国际舞台发出了景德镇声音。顺利举办第14届国际陶瓷博览会，参会企业档次、产品展示水平、会展交易额和国际影响力进一步提升。成功承办全省“双修”、创卫两个现场会，前所未有。九景衢铁路通车，高铁商务区框架基本建成，开启了景德镇“连接东西、沟通南北”的新时代。全长近30公里的地下综合管廊实现当年开工当年基本建成，创造了新的“景德镇速度”。

——这一年，我们力推了一项项重大改革。城管体制改革迈出历史性步伐，启动实施城乡环卫一体化、园林市场化改革和“公安+城管”联勤执法试点工作；积极推动首部地方性法规《市容和环境卫生管理条例》颁布实施；改革了市区两级财税体制，为加快建立现代财政制度、规范市区两级税收征管秩序提供了有力支撑；出台了加强我市中心城区农民住房建设管理的意见，引导农民逐步迁入小区、融入社区，城市化进程进一步加快。

一年来，我们主要抓了六个方面工作：

（一）全力开展环境整治，推动城市建设有颜值更有品质。全城动员、全民参与、全域行动，开展声势浩大的城乡环境大整治行动，“啃硬骨”、治顽疾、去沉疴，彻底改变了城市面貌，告别了“脏乱差”历史，并向城市“双创双修”纵深推进。规划体系进一步完善，基本实现中心城区控规全覆盖，编制完成绿化、学校、燃气等10余项公共服务设施专项规划，我市被列为省级城市设计试点。老城区保护扎实推进，御窑厂遗址被国家文物局纳入申报世界文化遗产预备名单，御窑博物馆完成主体工程，启动18个遗产点本体保护工作。昌南拓展区核心区已具雏形，15条主次干道全面建成，四馆即将投入使用。景南、景北高速收费站完工；西河水系治理完成一期工程，建成近千亩新昌南湖和西河湾湿地公园；国家森林公园、昌江百里风光带浮梁段、宝石码头等成为市民休闲新去处。既重“面子”更重“里子”，改造背街小巷239条，新增绿地100多万平方米。完成棚户区改造13126户，较去年增长近一倍。强力打击“两违”，城区拆除违法建筑49万平方米，比2016年接近翻番；农村拆除“空心房”和违章建筑200多万平方米，超过前11年总和。

（二）大力实施转型升级，推动产业发展有实力更有活力。持续推进一、二、三产融合发展，不断壮大“3+1”产业。陶瓷产业向创意产业转型。引进洛可可公司打造洛客·景德镇陶瓷设计中心。名坊园二期基本建成。航空产业蓄势待发，高新区成为全省第一批军民融合产业示范基地。编制完成通航产业发展规划。航空小镇建设加快推进，路网全线贯通。昌飞公司加快融入国际航空产业链，与美国西科斯基等企业拓展合作领域，智能制造技术进入行业前列，昌飞直升机总装园竣工投产；江直公司建成培训中心、动力中心、总装厂房和国内首条滚床式直升机总装生产线，与乌克兰企业签署了合作组装生产直升机协议，全省首个直升机科技馆开馆；德利直升机完成厂房建设；航空科技园开工；22家企业落户航空零部件产业园并开工建设。汽车产业向全产业链迈进。北汽昌河12万台发动机项目破土动工，H40D、北斗星X5E等新车型上市；汽车配套产业园及海纳川产业园建设加快推进。化工产业历经环保洗礼，焕发新的生机。焦化、天新、富祥、世龙等企业主营业务收入均大幅增长，主要产品在国内外市场主导地位进一步巩固。同时，服务业水平持续攀升，增加值占GDP比重超过40%。全面启动创建国家全域旅游示范区工作，一批旅游项目进展顺利，旅游总收入529亿元，增长47.2%。电子商务持续快速发展，预计实现交易额318亿元，增长40%。连续4年获评全国电商“百佳城市”，昌江区石岭村获“中国淘宝村”称号。

（三）着力抓实三农工作，推动美丽乡村有形象更有素质。完成农村土地承包经营权确权登记颁证工作，农村综合产权交易平台建成投入使用。全面完成永久基本农田划定。新农村建设实现新跨越，围绕“一带三边五线”基本完成1000个新农村点建设。农田水利设施得到极大改善，全市8.8万亩高标准农田建设全面开工，建设蔬菜标准园3万余亩，新增茶园面积1万亩，实现造林面积2.3万亩，浯溪口水利枢纽工程快速推进，小型病险水库除险加固进入扫尾。奋力夺取了“6·23”抗洪抢险胜利。落实粮食安全责任制，粮食生产稳定增收，预计总产量66.2万吨。“乐平辣椒”成为国家地理标志农产品；“浮梁茶”跻身中国茶叶公用品牌价值30强。乡村发展潜力进一步迸发，乐平市乐港镇获评全国“一村一品”示范镇；浮梁县瑶里镇入选全国第二批特色小镇，严台村被评为中国最美休闲乡村。休闲观光农业等新型业态快速发展，全市现有国家级休闲农业示范点3个、全国五星级休闲农业与乡村旅游示范点2个、省级休闲农业示范点9个。脱贫攻坚扎实推进，工作机制进一步完善，在全省首创“产业+金融”扶贫模式，贫困人口精准退出5160人。

（四）强力推进环保整改，推动生态文明有理念更有效果。高度重视中央环保督察和环保部约谈，更加坚定了绿色发展的思想和行动自觉。扎实推进环保问题整改，成立9个专项整改小组，并举一反三出台全面整改方案，投入资金近8亿元，通过全面摸排找症结、精准施策强治理、重拳治违严监管、督察问责抓落实，集中治理了中心城区污水直排、乐平工业园、焦化集团等突出问题。针对扬尘、油烟、废气等实施大气污染防治行动，有力改善了空气质量。不断提升城市污水处理能力，全面完成西城区截污管网、老南河截污干网等项目，城区河流水质得到有效改善。大力推进国家生态文明试验区建设，制定行动计划，完成生态保护红线划定校核调整工作。进一步完善“河长制”工作机制，扎实推进乐安河流域重金属污染治理，加大水域河流监管与执法力度，关停了一批禁养区养殖场，完成水资源管理“三条红线”控制目标计划。全年空气优良率91.4%，全省第一，PM10平均浓度67微克/立方米，同比下降1.5%；PM2.5平均浓度41微克/立方米，下降4.7%；主要河流Ⅰ～Ⅲ类水质断面达标率91.7%。

（五）致力深化改革开放，推动发展空间有广度更有深度。持续推动“放管服”改革和“三十一证合一”、“一照一码”等商事制度改革，推行“双随机一公开”机制，强化事中事后监管。深入开展降成本优环境专项行动，累计为企业减负近12亿元。加快金融体制改革，成立政府性债务管理领导小组和财政金融工作协调小组，出台《政府性债务风险应急处置预案》，规范了举债融资行为。成立了PPP和政府购买服务工作协调小组，积极拓宽社会资本投资渠道。控房价、防风险等工作有序推进，房地产去库存成效明显。资源型城市“转型十年”成就展呈现了我市转型升级的历史巨变。招商活动成果丰硕。举办南京汽车产业推介会、武汉现代服务业恳谈会，积极参加赣港会、500强高峰论坛、央企入赣等重大招商活动，全年引进省外2000万元以上项目188个，增长3.9%；预计实际进资404亿元，增长10%。积极创建鄱阳湖国家自主创新示范区，加快推动科技成果转移转化。与北控集团、江西师范大学等建立战略合作关系。对外开放通道建设步伐加快，新开通景德镇至宁波和青岛两条航线，皖赣铁路改线工程进度过半，昌景黄高铁项目建设全面启动。

（六）倾力回应群众关切，推动民生事业有力度更有温度。持续增加民生投入，预计全年民生支出144.5亿元，占一般公共预算支出75.6%，比上年提高3.1个百分点。为民办10件实事的承诺基本兑现。城乡低保标准持续提高。新增城镇就业3.02万人、农村劳动力转移就业2.35万人，超额完成全年任务。鼓励支持创新创业，大学生陶瓷创业园、陶溪川邑空间等孵化基地快速发展。出台房屋征收补偿标准和实施办法等文件，大力推进依法征收；分配入住保障性住房3691套。持续深化公立医院改革，让群众得到极大实惠；充分依托院士工作站技术优势，医疗服务能力进一步提升。编制完成中心城区中小学布局规划（2016—2030年），全面启动9所学校建设。大力推进教育均衡发展，三个县（市、区）通过国家验收评估。市老年大学办学质量、办学水平不断提升，对外影响力进一步扩大。全力筹备第十五届省运会，12个体育场馆的新建、改造、维修工程加快推进。社会治安持续好转，群众安全感进一步提升。信访形势稳定，总量大幅下降。安全生产态势总体平稳，是全省唯一连续15年未发生重特大事故的设区市。

一年来，我们按照打造“民生政府、责任政府、高效政府、法治政府、清廉政府”的要求，坚定不移加强政府自身建设，扎实推进“两学一做”学习教育常态化制度化，牢固树立“四个意识”，严格落实中央八项规定精神，政府作风进一步转变。深入推进依法行政，自觉接受人大监督、政协监督和社会监督，办理人大代表建议202件，政协委员提案199件，办复率均为100%。加大行政监察、审计监督和政务公开力度，实施领导干部自然资源资产离任审计，党风廉政建设和反腐败工作向纵深推进。

各位代表，成绩来之不易，这是省委、省政府和市委正确领导的结果，是全市上下团结一心、奋力拼搏的结果，是各位市人大代表、政协委员和社会各界大力支持的结果。我代表市人民政府，向全市人民，向各民主党派、工商联、各人民团体和各界人士，向中央和省驻景单位、驻景部队和武警官兵，向关心支持景德镇发展的港澳台同胞、海外侨胞和国际友人，表示崇高的敬意和衷心的感谢！

同时，我们也清醒地认识到发展中还存在着不少困难和问题。主要是：经济总量不大、发展速度不快的问题仍然突出，产业发展缺少大项目好项目；城乡规划建设管理水平仍有待提高，基本公共服务仍然存在短板；社会治理存在薄弱环节，群众关注的一些热点、痛点问题尚未得到有效解决；政务环境有待进一步优化，少数公职人员还存在不作为、慢作为现象，履职能力、主动服务意识还有待进一步提高。对此，我们将坚持问题导向，采取措施努力克服解决。

二、2018年工作安排

2018年是贯彻党的十九大精神的开局之年，是改革开放40周年，是决胜全面建成小康社会、实施“十三五”规划承上启下的关键一年。做好今年的工作，意义重大。根据省委、省政府和市委的总体部署，今年政府工作的总体要求是：全面贯彻落实党的十九大、中央经济工作会议和省委十四届五次全会精神，以习近平新时代中国特色社会主义思想为指导，按照高质量发展的要求，按照市委十一届四次全会的部署，坚持稳中求进工作总基调，贯彻新发展理念，以供给侧结构性改革为主线，以“双创双修”为主战场，以项目建设为主抓手，以“铸魂”为重点，着力推进改革开放，着力彰显文化特色，着力实施乡村振兴战略，着力有效改善民生，全力实施“三个五”战略行动，进一步改进政府工作，奋力迈出谱写新时代中国特色社会主义景德镇篇章的坚实步伐。

2018年，全市经济社会发展的主要预期目标是：生产总值增长8.5%左右；财政总收入增长7.5%左右；规模以上工业增加值增长8.8%左右；固定资产投资增长12.5%左右；社会消费品零售总额增长12.5%左右；外贸出口增长3%左右；实际利用外资增长9%左右；城镇居民人均可支配收入增长8.5%左右；农村居民人均可支配收入增长9%左右；居民消费价格总水平涨幅控制在2.9%左右；节能减排完成省下达的计划任务。

为实现上述目标，我们将着力做好以下工作：

（一）坚持以百亿项目千亿产业为目标，在加快产业升级上寻求新动能。以陶瓷、航空、汽车、旅游“3+1”特色产业为核心，在全力振兴主导产业的同时，加快培育新业态新动能，力争尽快形成若干千亿产业集群，推动产业在转型升级中实现高质量发展。

壮大主导产业。坚定陶瓷产业发展信心，构建大陶瓷发展格局。多措并举支持手工制瓷发展，释放陶瓷产业发展活力；优化顶层设计，紧盯艺术陶瓷、高技术陶瓷和日用高端陶瓷市场，精准发力，抢占产业链制高点；推进总投资45亿元的邑山陶瓷工业综合体、红叶陶瓷新厂、陶瓷设计研发中心等20个项目建设；进一步加强陶瓷市场监管、知识产权保护，加快打造高端陶瓷交易、高科技陶瓷孵化、陶瓷产品鉴定平台；做好陶瓷产业补链延链工作，推动产业做大做强，力争早日实现陶瓷企业上市。抢抓政策机遇发展航空产业，深化与昌飞公司、602所合作，推进军民融合示范区建设，着力打造一流的航空特色小镇；推动总投资50亿元的航空零部件园、航空科技园等15个项目建设；积极融入国际直升机合作生产体系，争取引进一批整机和航空零部件企业落户；提升直升机总装园生产能力，力争实现直升机产能200架；不断丰富通航产业业态，拓宽通航领域市场。支持北汽昌河创新管理方式，优化营销网络布局；开工建设北汽昌河研发中心项目，瞄准新能源汽车市场，不断研发投产新车型；加大海纳川和汽配园招商力度，提高零部件企业本土配套比例，促进产业集聚。推动旅游管理体制改革，出台产业扶持政策，打造一批陶瓷文化游、特色工业游和生态乡村游等精品线路，加快建设国家全域旅游示范区。

培育发展新动能。推动现代服务业提质升级，重点以昌南拓展区、高铁商务区为载体，加快建设商业综合体、金融街等项目；大力发展现代物流业，培育引进一批技术领先、主营业务突出、带动力强的现代物流企业集团；加快发展电子商务，大力扶持本土电商平台发展，积极引进外地成熟电商。推进“众创业、个转企、小升规、规转股、股上市、创龙头、育集群”，着力发展一批专精新特小微企业。进一步拓展新兴消费领域，大力发展医疗、养老、教育、文化、体育等服务。合理布局消费网点，新增一批民生商业设施，创造更加便利实惠的消费环境。

优化园区平台。推进新城区和园区管理体制改革，优化高新区功能。进一步明晰园区定位，采取“飞地经济”、联合共建、委托管理等形式，形成各有特色、错位竞争的良性发展态势。支持高新区围绕千亿产业目标，重点发展航空产业和新兴技术产业；支持陶瓷工业园区重点发展陶瓷产业，力争产值突破300亿元；支持乐平工业园区优化“一园三区”格局，重点发展医药化工产业，力争产值突破500亿元。针对园区功能定位，实施产业链精准招商，加强和国内外知名企业、龙头企业、创新企业的对接合作，将园区打造成景德镇创新驱动的主引擎、产城融合的主平台、招商引资的主力军、经济发展的主战场。

（二）坚持以城市“双创双修”为抓手，在提升城市品位上再求新成效。深入推进生态文明试验区建设，全力实施城市“双创双修”，重点打造“一带两城”，着力开展“十大行动”，为城市“塑形铸魂”，争取“双修”修出美、“双创”创成功，努力建设一座精致精美之城。

提升城市形象。加强以昌江百里风光带建设为中心的自然生态保护与修复，全力做好“治山理水、显山露水”文章，打好蓝天净土保卫战，努力使瓷都天更蓝、水更清、山更美、土更净。以“一江三河六山”为核心，推进九皇宫、旸府山等一批山体修复项目，加快西河湿地公园二期、凤凰山公园、松涛公园、云门公园及一批街头绿化工程建设，力争新增公园绿地270公顷。加大入河排污口监管力度，强力整治城市黑臭水体。加快城镇污水处理厂和配套管网建设，提升污水处理率，新建三宝、银坑、历尧3个污水处理站，实施西瓜洲污水处理厂提标扩容、高新区道路雨污分流改造等一批工程。建立大气污染预警预报系统，强化网格化属地管理，加强重点行业企业大气污染物综合治理，扎实推进扬尘治理，确保空气质量保持全省前列。落实土壤污染风险管控制度，逐步推开垃圾分类工作，积极推行垃圾无害化处理，启动建筑垃圾处理厂建设，打造“清洁土壤”。积极开展碳汇评估，探索碳排放交易，努力将生态优势转变为生态效益。

补齐功能短板。突出规划引领，启动全域空间规划编制，促进“多规合一”；加快“双修”规划编制，提升城市设计水平；继续保持打击“两违”高压态势。加快功能新区建设，昌南拓展区合理布局金融、商业综合体等功能设施建设；高铁商务区加快土地收储工作，建设一流高铁交通枢纽；坚持市民需求导向，全力打造15分钟便民生活圈和15分钟绿色休闲圈。加快做好地下综合管廊管线入廊和运营管理工作，实施一批城中村改造、道路延伸改造、环卫、防洪排涝等项目，加快推进昌景黄高铁、机场迁建等重大项目，构建外联内通的大交通格局。

全力开展“双创”。决战决胜创建全国文明城市、国家卫生城市，不断提升城市内在凝聚力、亲和力和外在竞争力、辐射力。加快推进“雪亮工程”，积极联网整合数字城管、国土“一张图”等系统，建立完善智慧城市平台，提高城市数字化、精细化管理水平。加强社区建设和管理，推动新建小区、“三无小区”及老旧小区建立物业自治管理机制，优化社区设置，强化社区功能，让社区成为最活跃的“城市细胞”。持续开展文明城市、村镇、单位、家庭、校园等创建活动，推动精神文明建设向更深层次、更广范围迈进。实施街面整治行动，全面实行“包卫生、包绿化、包秩序”“门前三包”制度，加大对主次干道、重要街区、学校、小区等公共场所周边流动摊点整治规范力度。围绕市民普遍关心的健康热点问题加强宣传教育，普及卫生健康知识。力争全国文明城市测评进入全省前三名、基本达到国家卫生城市标准。

（三）坚持以乡村振兴战略为引领，在推进农村繁荣上力求新进展。优先满足要素配置、优先保障资源条件、优先安排公共服务、优先发展农业农村，让农业成为有奔头的产业、农民成为有吸引力的职业、农村成为生态宜居的家园。

大力发展现代农业。始终把“产业兴旺”作为乡村振兴战略的核心，坚持在文化与生态交汇处做大优势产业、在传承与创新结合上做好特色产业、在一产与多产融合中做强高效产业。加强农业水利和高标准农田等基础设施建设，落实好第二轮土地承包到期后再延续30年政策，进一步完善粮食储备体系，大力拓展粮食贸易、发展粮食产业，夯实农业发展基础。立足“乐平菜”、“浮梁茶”，引导更多社会资本、技术、人才等要素汇集农业农村，加快实现“园区变景区、田园变公园、产品变礼品、农房变客房”。多措并举培育一批高素质农业生产职业队伍和新型农业经营主体，大力发展农产品加工业、流通业和休闲农业，推进农业产业化经营。积极支持焦化集团和浮梁县推进“高岭·中国村”田园综合体项目，探索建立集“现代农业、休闲旅游、田园社区”为一体的乡村综合发展模式。加快现代农业示范园区建设，支持乐平国家级农业科技示范园建设国家一、二、三产融合发展试验区，提高农业产业化水平。

扎实开展美丽乡村建设。以“六统五美四联创”为抓手，建设美丽集镇“133”工程，着力打造以陶瓷文化艺术、古村落文化、乡村生态旅游为特色的样板村，实现由“村容整洁”向“生态宜居”升级。加快推进青山白化整治工程，积极倡导绿色殡葬。全面实施城乡环卫全域一体化第三方治理，创建景瑶线、乐弋线等美丽风景示范线，推动美丽乡村建设由点到面、由面到片，将一个个“盆景”打造成一幅幅靓丽“风景”，由产业依赖转变为生产生态、生活生态、人文生态、环境生态并重，成为产、镇、人、治兼备的乡村生活新载体。

深入推进脱贫攻坚。严格按照脱贫目标，聚焦“两不愁、三保障”，针对贫困户发展不起来、看不起病、上不起学、住不起房等突出问题，建立全覆盖的精准扶贫、监测评估、防范返贫、责任落实四项机制，扎实推进精准扶贫、精准脱贫。坚持“扶贫+扶志+扶智”，激发脱贫发展内生动力，防止“养贫助懒”。深入实施脱贫攻坚十大工程，扩大“产业+金融”扶贫成果，推动产业扶贫全覆盖，提高贫困群众脱贫致富能力，改善贫困村生产生活条件，确保5个省级贫困村摘帽、6073人脱贫。

（四）坚持以老城保护为重点，在增强文化软实力上务求新提升。全力创建景德镇陶瓷文化传承创新示范区，厚植文化根脉，铸好文化之魂，让传统陶瓷文化更有魅力、新兴文化创意产业更具活力，为繁荣兴盛中国文化贡献“景德镇力量”。

传承保护历史文化。以最优的理念、最严的规划、最高的标准、最好的质量、最佳的业态实施历史文化名城保护，重点推进陶阳里及御窑厂遗址保护项目，力争早日成为世界文化遗产。推动御窑厂遗址保护完成立法工作。继续实施老厂区、老里弄、老窑址保护，全力推进三闾庙历史文化街区改造等项目。加大文化遗产资料收集抢救及传统制瓷技艺保护传承，让历史文化遗存传下去、活起来。

加快发展文化产业。深入贯彻“文化＋”发展理念，加快建设一批重大文化产业项目、提升一批重点文化产业园区、培育一批文化创意产业龙头企业，推动以陶瓷为主导的文化产业提速发展。重点抓好名坊园、陶溪川二期、三宝瓷谷三期等项目建设。启动陶瓷大学创意小镇项目建设，打造集陶瓷设计、材料研发、交流展示、创意博物馆于一体的创意创新创业平台，建成创意陶瓷总部经济基地。推动文化类企业做强，支持中小微文化企业、个体创作者、创客空间等发展。抓好知名文化品牌建设，走陶瓷品牌市场化运作之路。

扩大文化交流合作。以陶瓷文化为桥梁，充分发挥“景漂”“景归”作用，加大走出去步伐。深化与故宫博物院、中央美术学院、中国美术家协会等合作，积极携手中国艺术研究院等机构，进一步扩大陶瓷文化影响力。高水平办好瓷博会，不断提升文化交流层次与内涵。积极融入全球创意城市网络、海上丝绸之路城市联盟，广泛开展对外交流并承办相关年会活动，推动陶瓷文化在交流中互鉴、互鉴中融合、融合中创新、创新中发展。

（五）坚持以深化改革开放为动力，在创新体制机制上谋求新机遇。坚决革除一切僵化保守的思想观念和体制机制弊端，提高改革推动力、开放带动力、创新驱动力，为经济社会发展注入强大动能。

攻坚重点领域改革。（1）深化供给侧结构性改革，抓好“三去一降一补”，积极发挥市场在资源配置中的决定性作用，切实提高供给体系质量。（2）全面推开“一次不跑”改革，大力推行“互联网+政务服务”，打造“放管服”改革“升级版”。（3）深化商事制度改革，实施市场准入负面清单制度，打造公平竞争的市场环境，激发各类市场主体活力。（4）推进高新区体制机制改革，做优产业发展平台，使园区真正成为创新引领区、开放先行区、改革试验区、合作示范区和重要增长极。（5）深化国企国资改革，进一步完善公司法人治理结构，着力转变国资监管职能，优化国有经济布局结构，做大做强做优国有资本。（6）深化金融体制改革，加强金融债务管理和融资平台监管，以增量盘活存量，在防范负债风险、维护金融市场稳定的前提下，积极引导和规范发展新型金融业态。（7）深化人才体制机制改革，进一步完善人才政策体系，提高“景漂”“景归”人才服务水平，出台更具含金量的举措，实现人才集聚、蜂拥、叠加效应。

扩大对外开放合作。积极参与“一带一路”建设，推动产业、资本、人才等要素全方位开放。瞄准产业链开展专业招商和以商招商，建立健全招商项目综合评估制度，完善从项目评估、洽谈、签约到开工、建设、投产等各环节的保障推进机制，缩短项目落地周期。举办形式多样产业推介活动，鼓励社会资本进入现代物流、电子商务、金融服务、健康养老、医疗卫生、城市综合体等现代服务业领域。

深入推进创新创业。全面深化与浙江大学、北京航空航天大学、江西师范大学等高等院校战略合作，支持景德镇陶瓷大学、景德镇学院等加强科研攻关，打造科技创新平台和团队，加快科技成果产业化。继续实施创新驱动“54321”工程，力争新增10家高新技术企业、8个科技研发与服务平台，实施6个科技创新重大专项，培养引进4个科技创新领军人才及团队，取得2项重大科技成果。倡导创新文化，强化知识产权创造、保护、运用。推进大众创业万众创新，营造优良创业环境，释放更强创业活力。

（六）坚持以人民安居乐业为基石，在增进民生福祉上追求新发展。始终坚持以人民为中心的发展思想，加快补齐民生短板，注重提升民生内涵，尽最大努力满足人民群众对美好生活的向往。

着力办好民生实事。按照“群众提、大家定、政府办”的理念，今年继续为群众重点办好十件实事。（1）购买2600个公益性岗位，帮助困难群体就业；发放小额创业贷款4.35亿元，促进创新创业；发放一次性求职创业补贴400人次，支持大学生就业创业。（2）完成10000户城市棚户区改造。（3）建成10条全民健身路径。（4）完成102所市级贫困村标准化卫生计生服务室建设。（5）购置40辆纯电动公交车，新开通2条市内旅游公交线路及3条高铁商务区公交专线，优化调整8条公交线路，加密城市主干道公交班次。（6）改造提升170座公厕（含旱厕），新建4座垃圾中转站。（7）对43条主次干道进行局部升级改造。（8）续建新建10个中心城区停车场，新增2200个停车泊位。（9）完成西路、里村、星荷湾、西路新区、方家山5个城区农贸市场改造；启动新建古城、朝阳路2个城区农贸市场；完成乐平市湾头、南港及浮梁县鹅湖3个县乡农贸市场改造项目。（10）新建10个城乡居家养老服务中心（站）。

统筹发展社会事业。当好东道主、喜迎八方客，举全市之力承办好一届体现景德镇特色的高水平省运会。合理配置教育资源，统筹推进城乡教育均衡发展，继续推进陶阳学校、昌江区实验学校等9所学校建设。加强和规范幼儿园管理，从严治理中小学补课、托管等乱象。积极推动公共文化服务标准化、均等化，加快完善现代公共文化服务体系，实施文化惠民工程，广泛开展群众性文化活动，启动城市规划馆、青少年宫、科技馆及社区综合文化站等建设，推动公共文化服务向社区和农村延伸。持续开展“健康瓷都”建设，继续深化城市公立医院综合改革，提升基层医疗卫生机构服务能力。

不断创新社会治理。加强人民调解与行政调解、司法调解衔接配合，完善多元矛盾化解体系。推进法治信访、阳光信访和责任信访，及时解决群众合理诉求。持续深化“平安瓷都”建设，加强社会治安综合治理，创新立体化社会治安防控体系，推动群众安全感满意度“双提升”。加强重点建设、重大项目风险评估，在注重经济效益的同时切实维护社会稳定。加强应急管理体系建设，提升防灾减灾能力。推进安全生产领域改革发展，严格落实安全生产责任制，坚决遏制重特大事故发生。凝聚军地合力，抓好国防后备力量建设，维护军人军属合法权益。同时，支持工、青、妇、红十字会等群团组织发挥更大作用，扎实做好审计、机关事务管理、民族宗教、外侨、对台、人防、统计、档案等工作。

各位代表！落实党的十九大各项战略部署，完成今年各项目标任务，对政府自身建设提出了更高要求。我们将不忘初心牢记使命。强化“四个意识”，提高政治站位，增强政治自觉，坚决维护党中央权威和集中统一领导，坚决维护习近平总书记在党中央、全党的核心地位，坚决贯彻党中央、国务院、省委、省政府和市委的各项决策部署，确保政令畅通、令行禁止。我们将不负重托勇于担当。以实绩论英雄，以实干促发展，建立正向激励机制，为敢于担当者担当、为敢于负责者负责，使勇担当、敢作为蔚然成风。我们将不遗余力狠抓落实。坚决反对形式主义、官僚主义，始终把狠抓落实作为政府工作的主旋律，以“钉钉子”精神一抓到底、抓出成效。我们将不逾底线廉洁奉公。严格落实全面从严治党主体责任，严格遵守中央八项规定实施细则精神，落实“一岗双责”，加大整治“四风”问题力度，认真对待群众反映强烈的问题，坚决查处损害群众利益的行为，维护清正清廉清明的人民政府形象。我们将不留盲区接受监督。加快法治政府建设，严格按照法定权限和程序行使权力、履行职责。认真执行人大决定，自觉接受人大及其常委会、政协、监察、社会、舆论等各类监督。

各位代表！新思想引领新时代、新时代开启新征程、新征程要有新作为。我们无比欣喜地看到，景德镇的发展又站到了新的历史起点上。我们要更加紧密团结在以习近平同志为核心的党中央周围，以习近平新时代中国特色社会主义思想为指导，在省委、省政府和市委的坚强领导下，全力实施“三个五”战略行动，抢抓机遇，奋发作为，为打造一座与世界对话的城市、奋力谱写新时代中国特色社会主义景德镇篇章而不懈奋斗！
